# Supplementary material for: Vertical optokinetic eye movements in the larval zebrafish
Source: J Exp Biol. 2026 Apr 9;229(8):jeb251770. doi: 10.1242/jeb.251770 (PMC13091498; doi:10.1242/jeb.251770)
Supplement: Supplementary information [file jexbio-229-251770-s1.pdf]

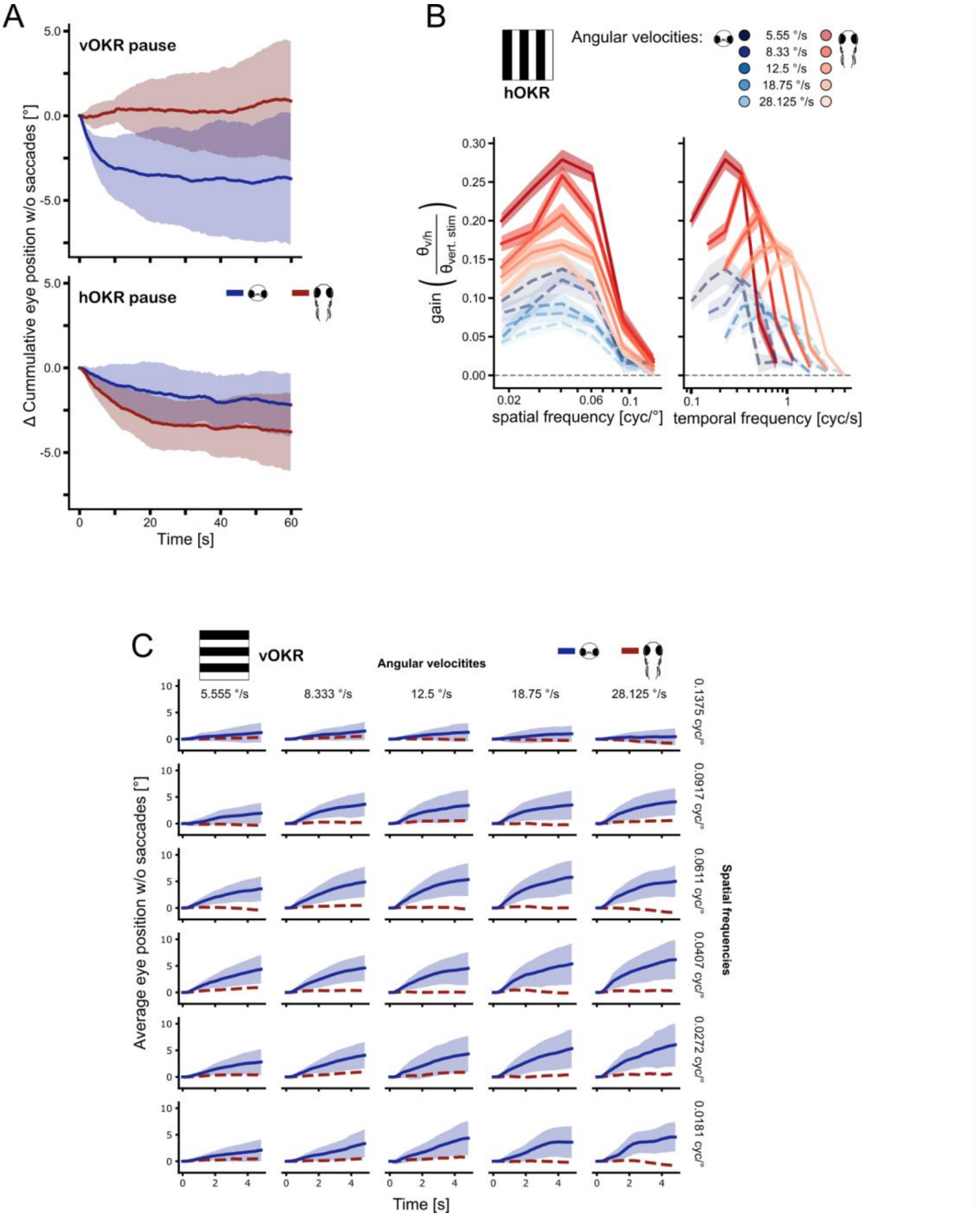


**Fig. S1. Additional data for constant rotation OKR stimulus analysis. (A)** Relative Average cumulative eye position with saccades removed for pause breaks during vOKR (top) and hOKR (bottom) stimulus protocols. Depending on the rotation direction of the previous stimulation, the individual eye traces have been inverted. **(B)** Gain tuning to spatial frequency and temporal frequency for all different angular velocities. Lines show mean and standard error of the mean. Red: gain for horizontal eye trace for hOKR stimulation. Blue: gain calculated for vertical eye trace for hOKR stimulation. Dashed horizontal line indicates a gain of 0. **(C)** Average start response during the first seconds of vOKR stimulation for all stimulus combinations. Blue line indicates average vertical eye trace and envelope indicates standard deviation. Red dashed line indicates the average horizontal eye trace. n = 10.

Journal of Experimental Biology • Supplementary information

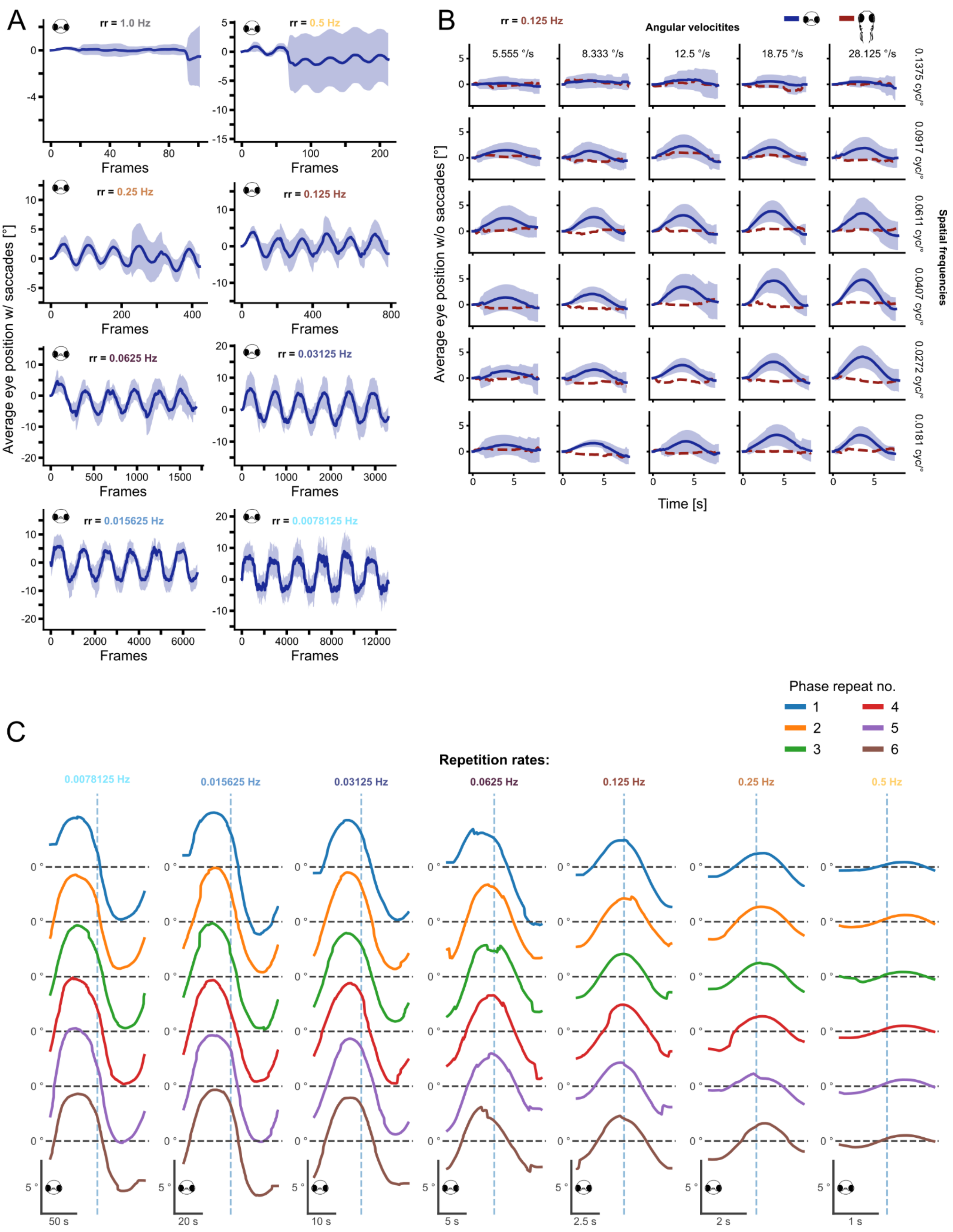


**Fig. S2. Additional Data for sinusoidal vOKR. (A)** Averaged eye positions for the entire stimulation with each repetition rate (rr) in the rr-tuning sinusoidal vOKR stimulus without saccade removal. First responses are elicited by rr = 0.5 Hz. For rr = 0.0625 Hz and lower, the eyes reach a maximum deflection which becomes more distinct for lower rr. Increase in standard deviation for 1.0, 0.5, and 0.25 Hz is due to individual fish that performed a large saccade during the stimulation and thereby shifted the eye trace into one direction. n = 8. **(B)** Average response to the stimulus (stimulus-triggered average, STA) for all stimulus

combinations of the fs-tuning sinusoidal vOKR stimulus. Blue line indicates vertical STA and envelope indicates standard deviation. Red dashed line indicates the horizontal STA. n = 11. **(C)** Individual average responses to each stimulus repetition for the different repetition rates of the rr-tuning vOKR stimulus. Dashed vertical line indicates midpoint of one stimulation phase as reference. Phase lag is consistent over all repeats within the same stimulus condition. n = 8.

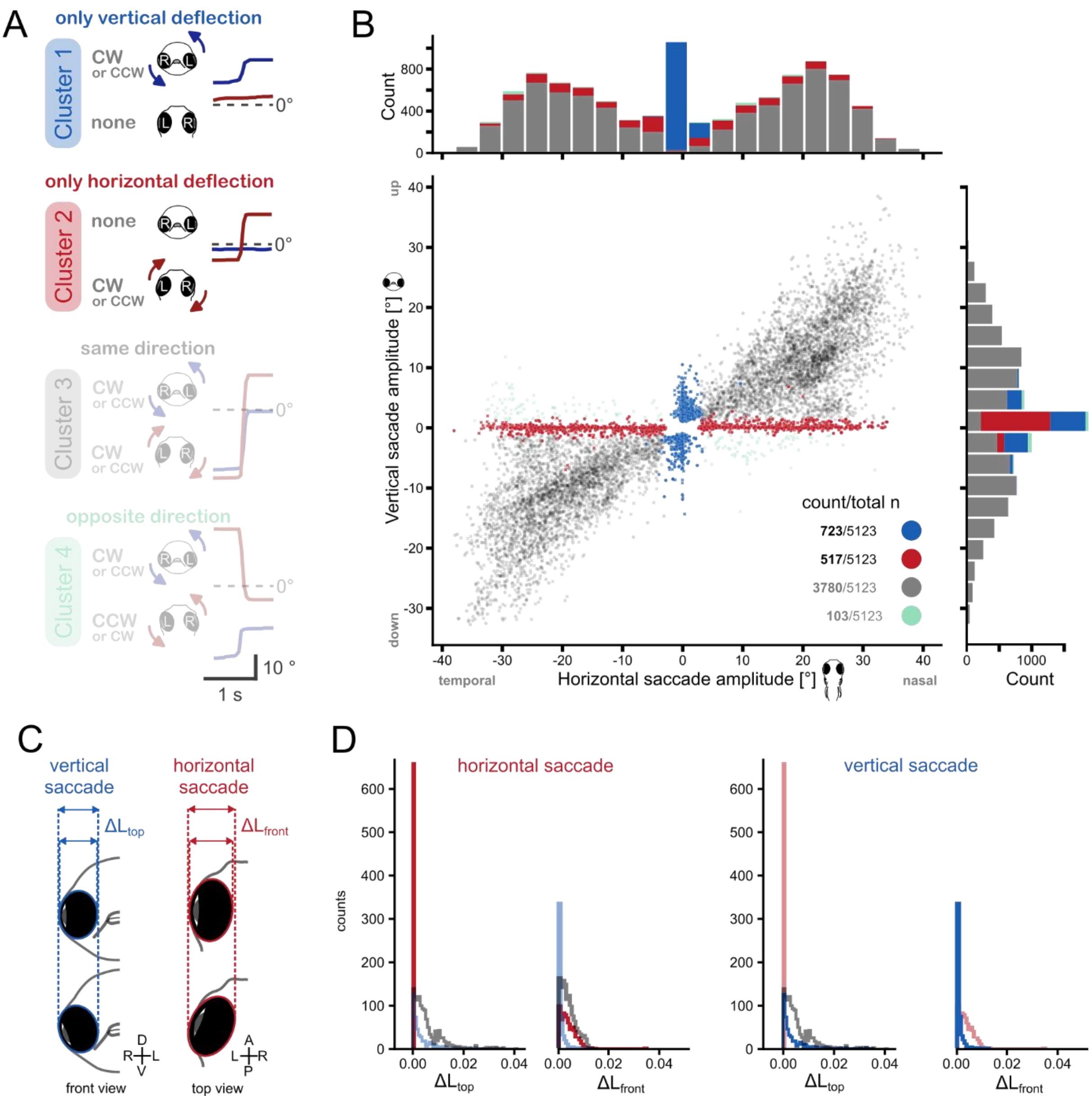


**Fig. S3. Saccade Clusters during vertical OKR (vOKR) stimulation. (A)** Four different saccade types have been observed and can be distinguished by their appearance in the vertical and horizontal eye trace. Example vertical (blue) and horizontal (red) eye traces show characteristics of each cluster type. Scale on the right corresponds to all eye traces. Note, that cluster 3 and 4 contain both mixed (vertical-horizontal) and pure horizontal saccades. Pure horizontal saccades can cause projection artifacts on the front camera (due to large movement, illumination and eye position) which can result in an apparent vertical movement. Cluster 3 and 4 were therefore not further analyzed. **(B)** Scatterplot of apparent horizontal and vertical amplitudes of all observed saccades, clustered by the saccade type during vOKR stimulus. **(C)** Illustration of the minor axis length change (after ellipse fitting) during pure vertical and horizontal saccades. **(D)** Distribution of minor axis length changes during horizontal (red) and vertical (blue) saccades for both top ($\Delta L_{front}$) and front ($\Delta L_{top}$) view. Red and blue bars represent the minor axis length change

distributions during horizontal and vertical saccades. Grey bars represent distributions during mixed saccades. During a horizontal saccade, the minor axis length changes of the top view are ~0 whereas the minor axis length changes of the front view are >0 (left). For vertical saccade, it is exactly the opposite (right). This measurement confirms detection of true horizontal and vertical saccades, as the minor axis length of the top view ($L_{front}$) should not change during a horizontal saccade, but should change during a vertical saccade and vice versa for the minor axis length of the front view ($L_{top}$).

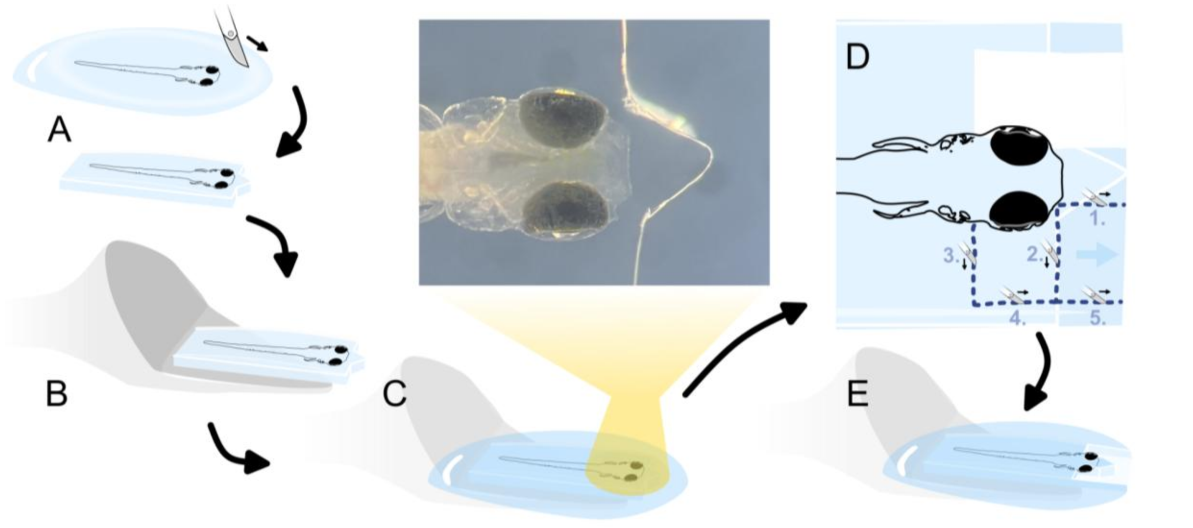


**Fig. S4. Schematic of zebrafish larva embedding procedure. (A)** The larva is immobilized in 1.6 % agarose and a block containing the fish is cut out. The image shows the triangle shape of the first agarose block in front of the head of the fish. **(B)** The block containing the fish is transferred onto a modified pipette tip. **(C)** The agarose block with the zebrafish is fixated on the pipette tip with agarose. **(D)** Scheme of steps to remove the agarose around the eyes. **(E)** The fish is ready for recording; the eyes can move and have no agarose in front of them while a small block of agarose remains in front of the head to prevent the fish from freeing itself.

Journal of Experimental Biology • Supplementary information

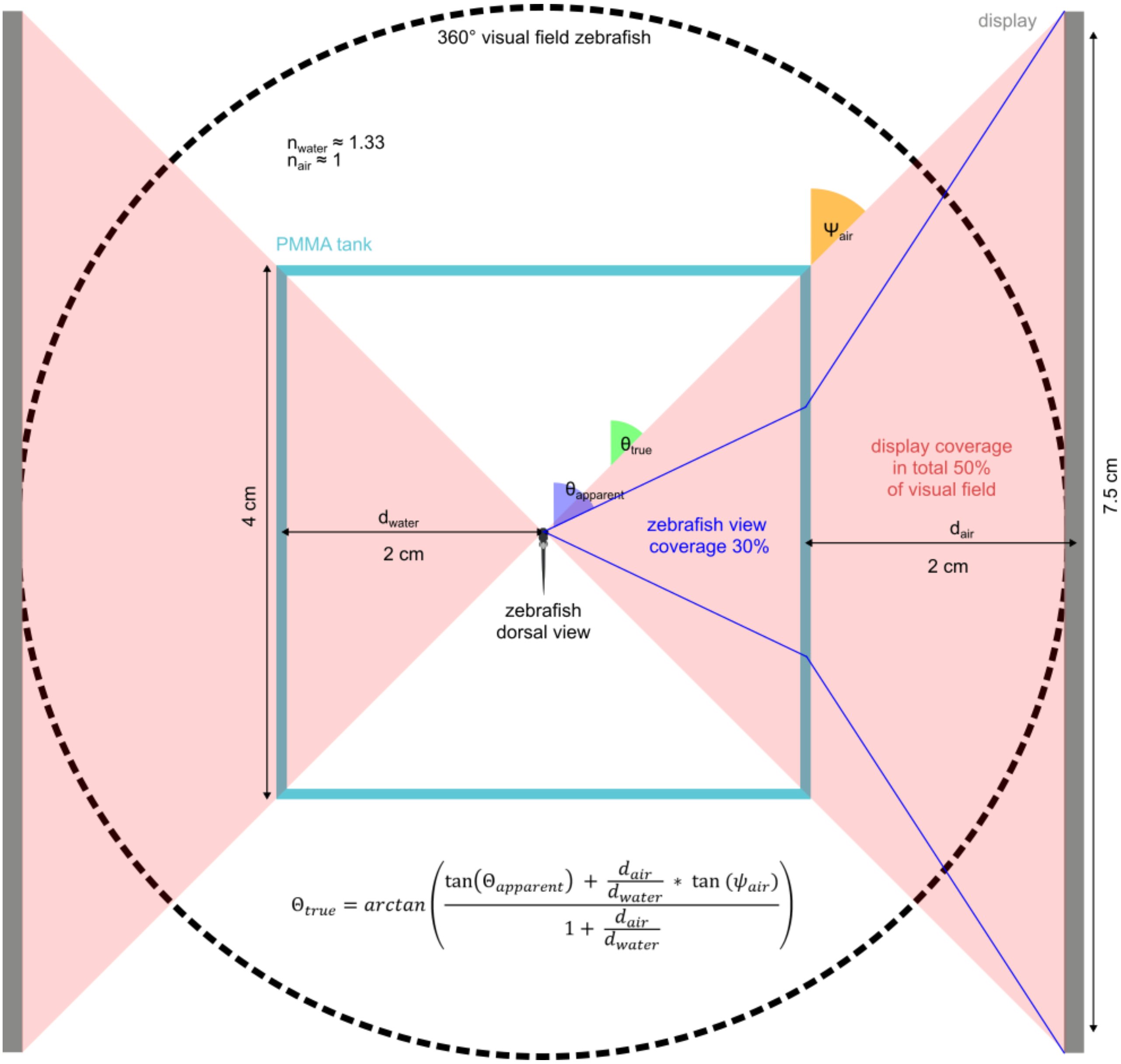


**Fig. S5. Dimensions and visual coverage of OKR setup. (A)** Dimension of the visual stimulation setup. Visual coverage of the arena was calculated according to Dunn and Fitzgerald (2020). Red area marks the visual coverage of the displays without correcting for physical distortions. Blue lines mark the area of visual coverage after correcting for refractive distortions.

Journal of Experimental Biology • Supplementary information

**Table S1. Stimulus parameters for assessing OKR tuning to spatial frequency and angular velocity.** Each stimulus parameter pair was repeated for both rotation directions. Parameter values were adapted from (Dehmelt et al., 2021). The order of the stimulus parameter pairs was randomized.

| Spatial Frequency [cycle/°] | Spatial Period [°/cycle] | Angular Velocity [°/s] | Temporal Frequency [cycle/s] |
|---|---|---|---|
| 0.0181 | 55.2486 | 28.125 | 0.77 |
| | | 18.75 | 0.51 |
| | | 12.5 | 0.34 |
| | | 8.333 | 0.23 |
| | | 5.555 | 0.15 |
| 0.0272 | 36.7647 | 28.125 | 0.77 |
| | | 18.75 | 0.51 |
| | | 12.5 | 0.34 |
| | | 8.333 | 0.23 |
| | | 5.555 | 0.15 |
| 0.0407 | 24.5700 | 28.125 | 1.14 |
| | | 18.75 | 0.76 |
| | | 12.5 | 0.51 |
| | | 8.333 | 0.34 |
| | | 5.555 | 0.23 |
| 0.0611 | 16.3666 | 28.125 | 1.72 |
| | | 18.75 | 1.15 |
| | | 12.5 | 0.76 |
| | | 8.333 | 0.51 |
| | | 5.555 | 0.34 |
| 0.0917 | 10.9051 | 28.125 | 2.58 |
| | | 18.75 | 1.72 |
| | | 12.5 | 1.15 |
| | | 8.333 | 0.76 |
| | | 5.555 | 0.51 |
| 0.1375 | 7.2727 | 28.125 | 3.87 |
| | | 18.75 | 2.58 |
| | | 12.5 | 1.72 |
| | | 8.333 | 1.15 |
| | | 5.555 | 0.76 |

Journal of Experimental Biology • Supplementary information

**Table S2. Stimulus parameters for assessing vOKR repetition rate tuning.** The stimulus was presented two times for each repetition rate and with a randomized direction of the maximum angular velocity.

| Spatial frequency [cycle/°] | Spatial period [°/cycle] | Angular velocity [°/s] | Repetition rate [cycle/s] | Length for 6 sine period repetitions [s] |
|---|---|---|---|---|
| 0.0611 | 16.3666 | 12.5 | 3.0 | 2 |
| | | | 2.0 | 3 |
| | | | 1.0 | 6 |
| | | | 0.5 | 12 |
| | | | 0.25 | 24 |
| | | | 0.125 | 48 |
| | | | 0.0625 | 96 |
| | | | 0.03125 | 192 |
| | | | 0.015625 | 384 |
| | | | 0.0078125 | 768 |

Journal of Experimental Biology • Supplementary information

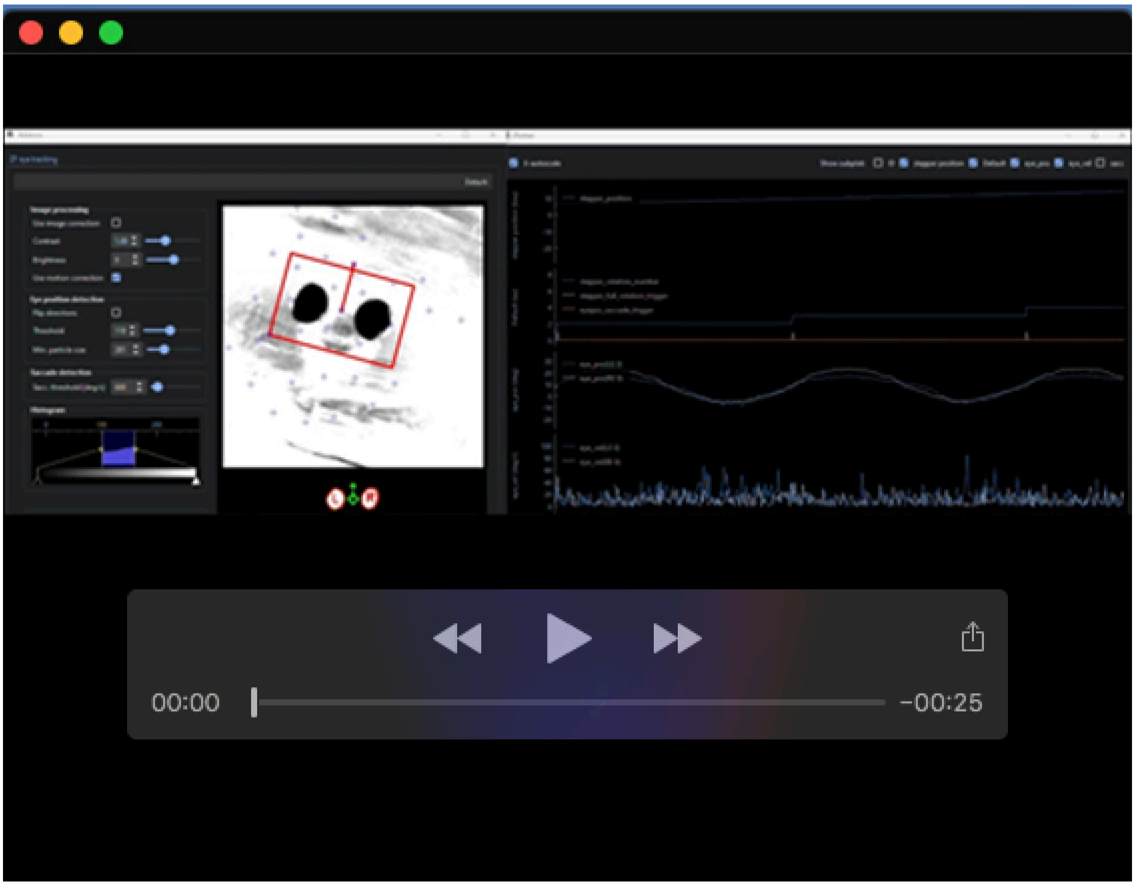


**Movie 1. Example recording of vVOR experiment.** Recording of vestibular stimulation protocol using the KEBAB setup with a velocity of 90°/s and constant rotation. The compensatory vertical eye movements are clearly visible in the camera (left side of video) and the corresponding eye traces of live tracking (using ellipse fitting on thresholded eye) display a sinusoidal pattern, visible in the right side. To ensure maximal eye tracking performance, image parameters like contrast and brightness were adjusted.

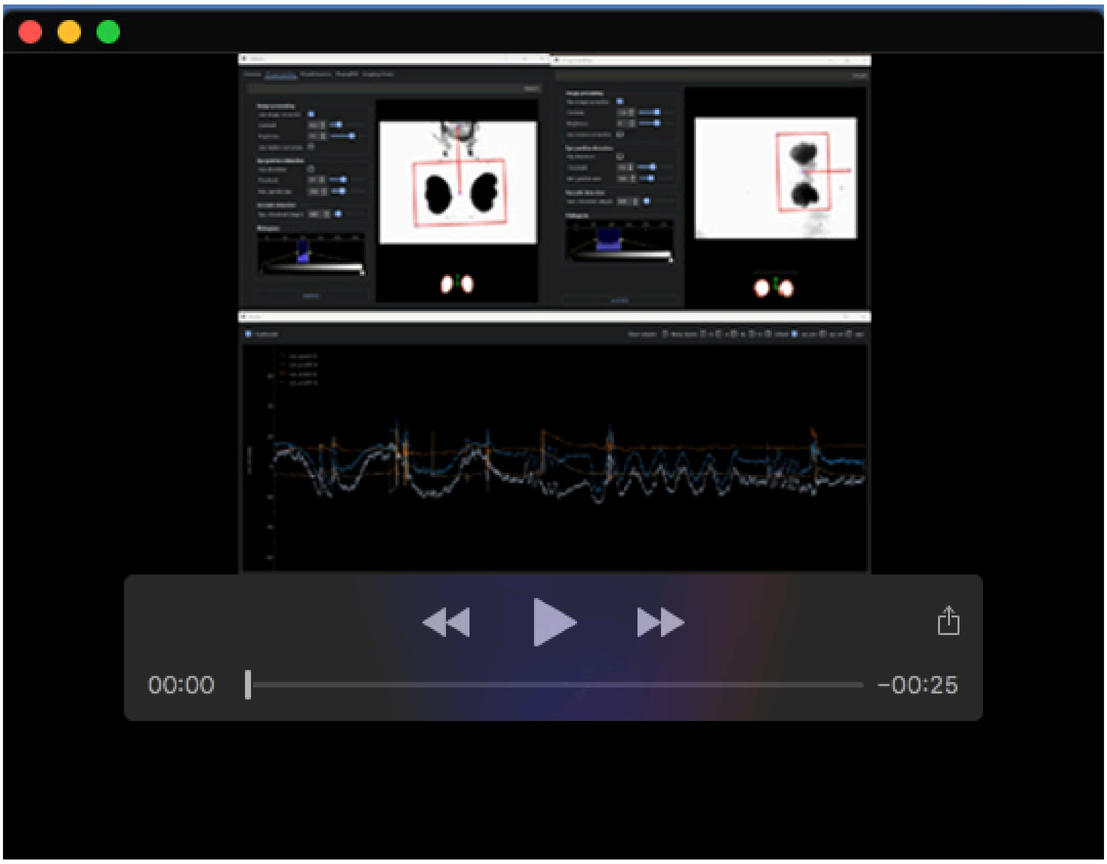


**Movie 2. Example recording of sinusoidal vOKR experiment.** Recording of sinusoidal visual stimulation using the two-camera visual stimulation setup. In the top half, both camera views (top and front) are visible. To ensure a good eye tracking performance, image parameters like contrast and brightness were adjusted. Note: The front image (right top corner) was rotated 90° due to the camera position. Pure vertical sinusoidal eye movements (vOKR) are clearly visible in both top and front camera view. The lower half shows the corresponding eye traces of live tracking. Bright and dark blue lines correspond to vertical eye movements and bright and dark orange lines correspond to horizontal eye movements. Note: The video was sped up to allow visual inspection of different phases with different repetition rates (as can be seen by the different frequencies of the sinusoidal vOKR).

Journal of Experimental Biology • Supplementary information

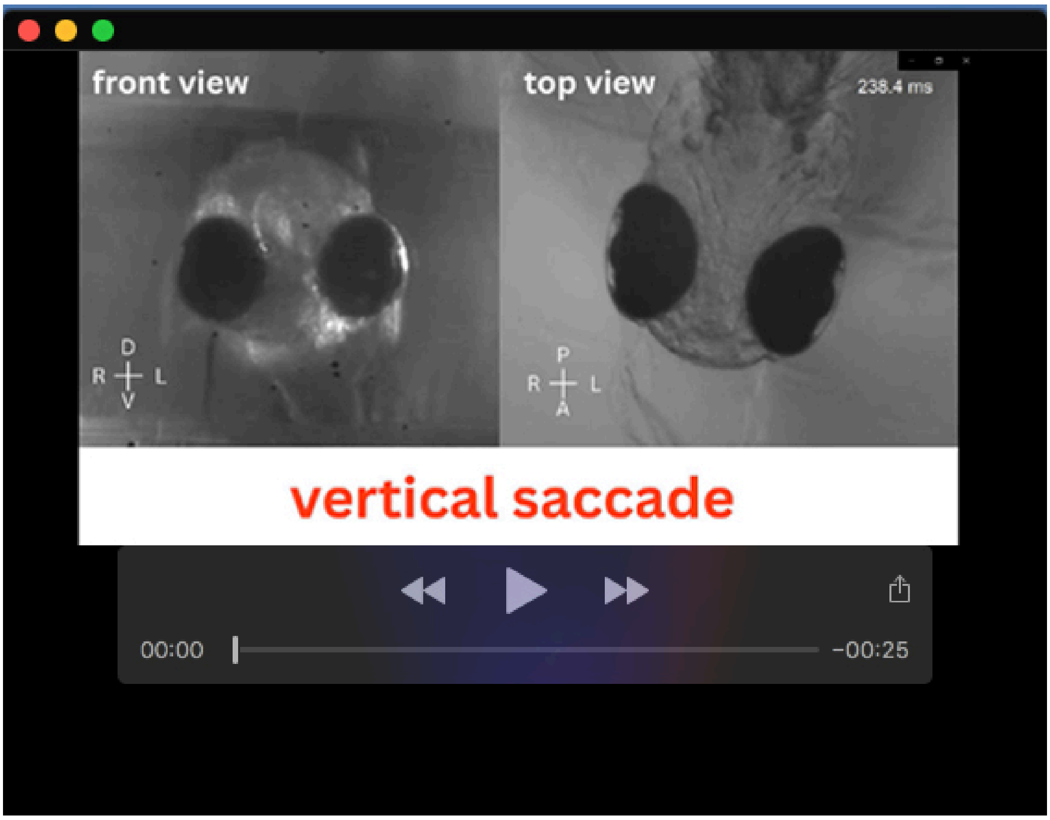


**Movie 3. Pure vertical saccade.** Video of front (left) and top (right) view during a pure vertical saccade.

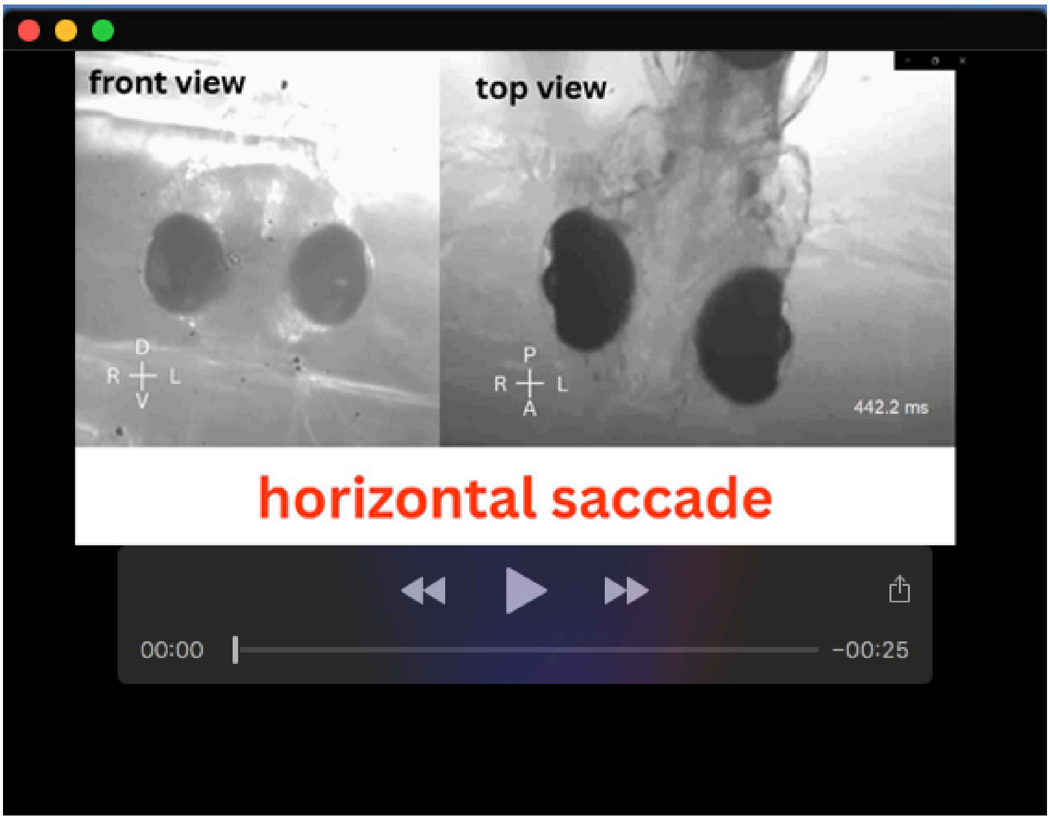


**Movie 4. Pure horizontal saccade.** Video of front (left) and top (right) view during a pure horizontal saccade.

Journal of Experimental Biology • Supplementary information

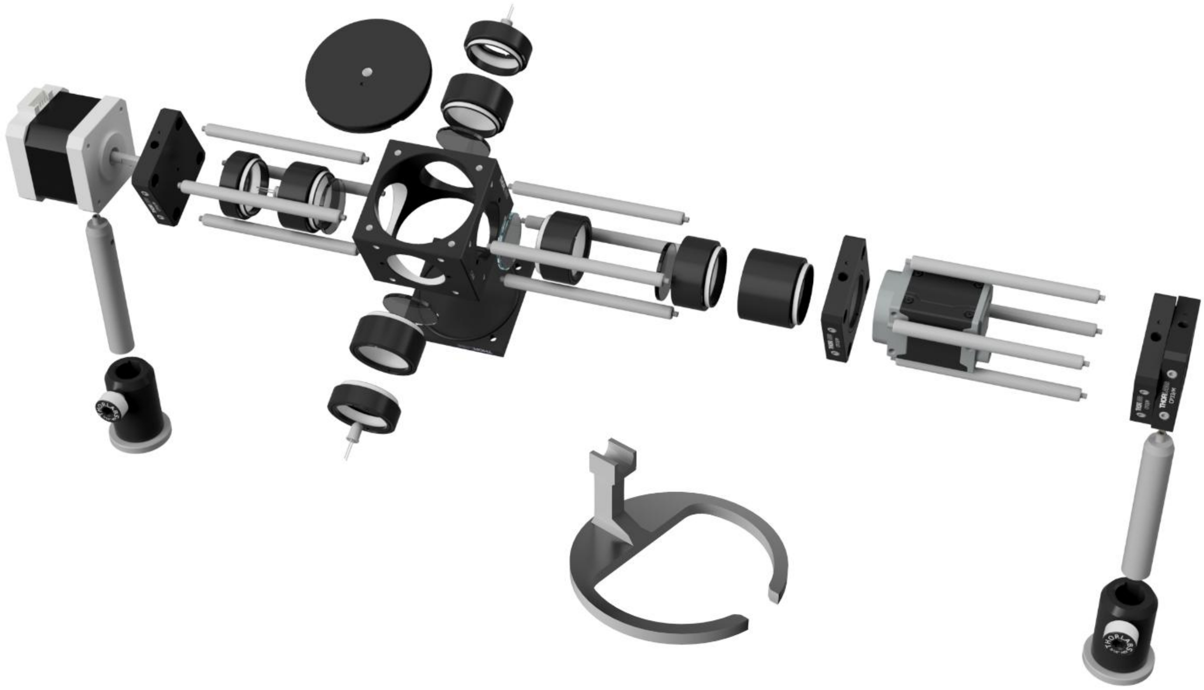

**Wiring Layout:**

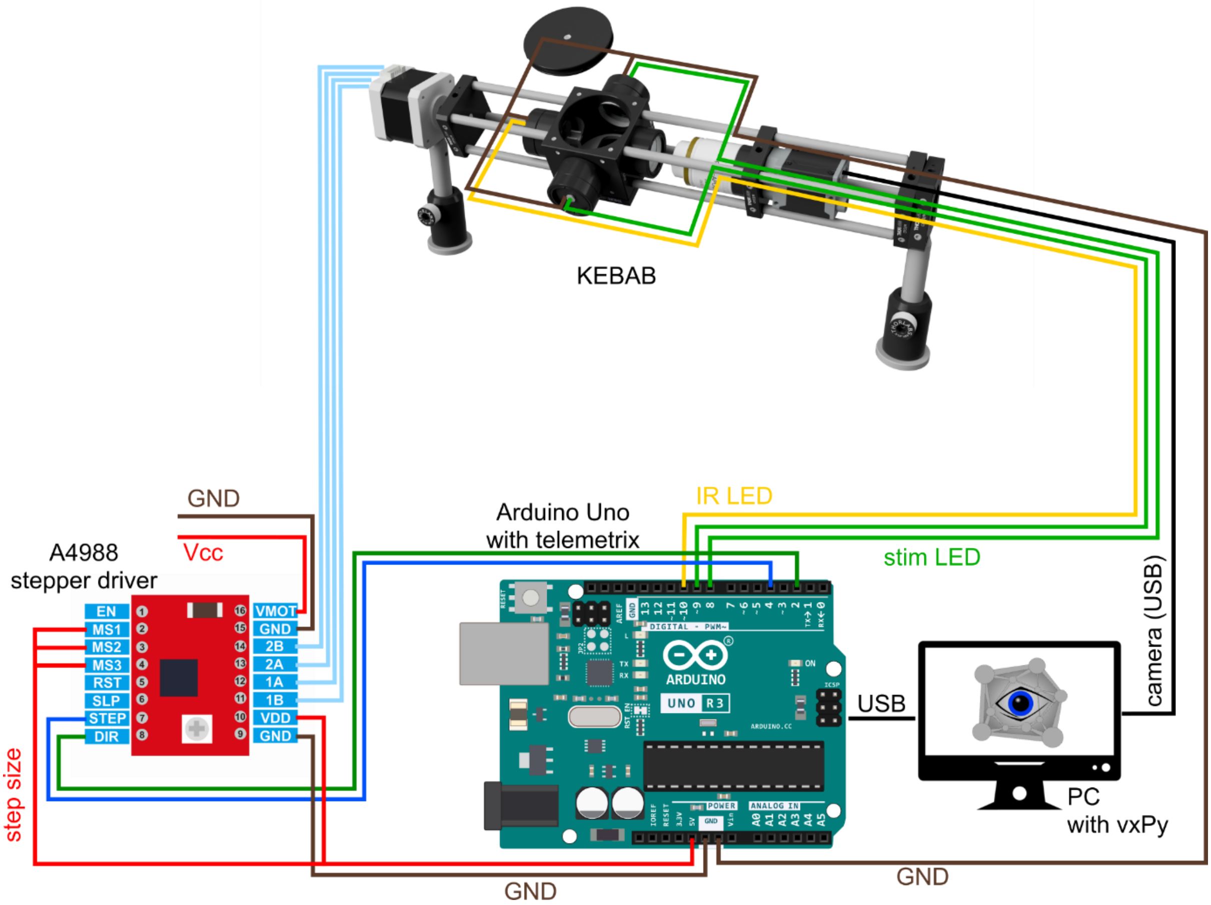

**Fig. S6.** KEBAB (Kinematic Evaluation of Behavior during Animal Body-roll)Construction Overview.

**Software:**

vxPy: https://github.com/thladnik/vxPy

telemetrix: https://mryslab.github.io/telemetrix/

**Components:**

1x NEMA 17 stepper motor, ACT Motor GmbH

5x SM1 Lens Tubes 0.5”, Thorlabs

3x SM1 Lens Tubes 0.3”, Thorlabs

3x 1” UV Fused Silica Ground Glass Diffusor, Thorlabs

1x 1” UVFS Broadband Precision Window, Thorlabs

1x 30mm Rotating Cage Segment Plate, Thorlabs

1x Blank 30mm Cage Plate with bore for NEMA 17 axle, Thorlabs

1x Blank Cover Plate, Thorlabs

1x 30 mm Cage Cube, Thorlabs

1x Fixed Cage Cube Platform for C4W/C6WR, Thorlabs

1x SM1-Threaded 30mm Cage Plate, Thorlabs

12x 3” Cage Assembly Rods for 30mm Cage, Thorlabs

2x 0.5” Optical Post, Thorlabs

2x 0.5” Pedestal Post Holder, Thorlabs

1x 0.48” Diameter 12 Wire Capsule Slip Ring, Amazon

1x IR LED 850 nm, e.g. Conrad

2x white LED, e.g. Conrad

1x 1” IR 850nm longpass filter, Thorlabs

1x camera DMK 23UV024, Imaging Source

1x C-mount 5x extender lense, e.g. bestscientific

1x Arduino Uno, Conrad

1x A4988 stepper driver, Conrad

Journal of Experimental Biology • Supplementary information

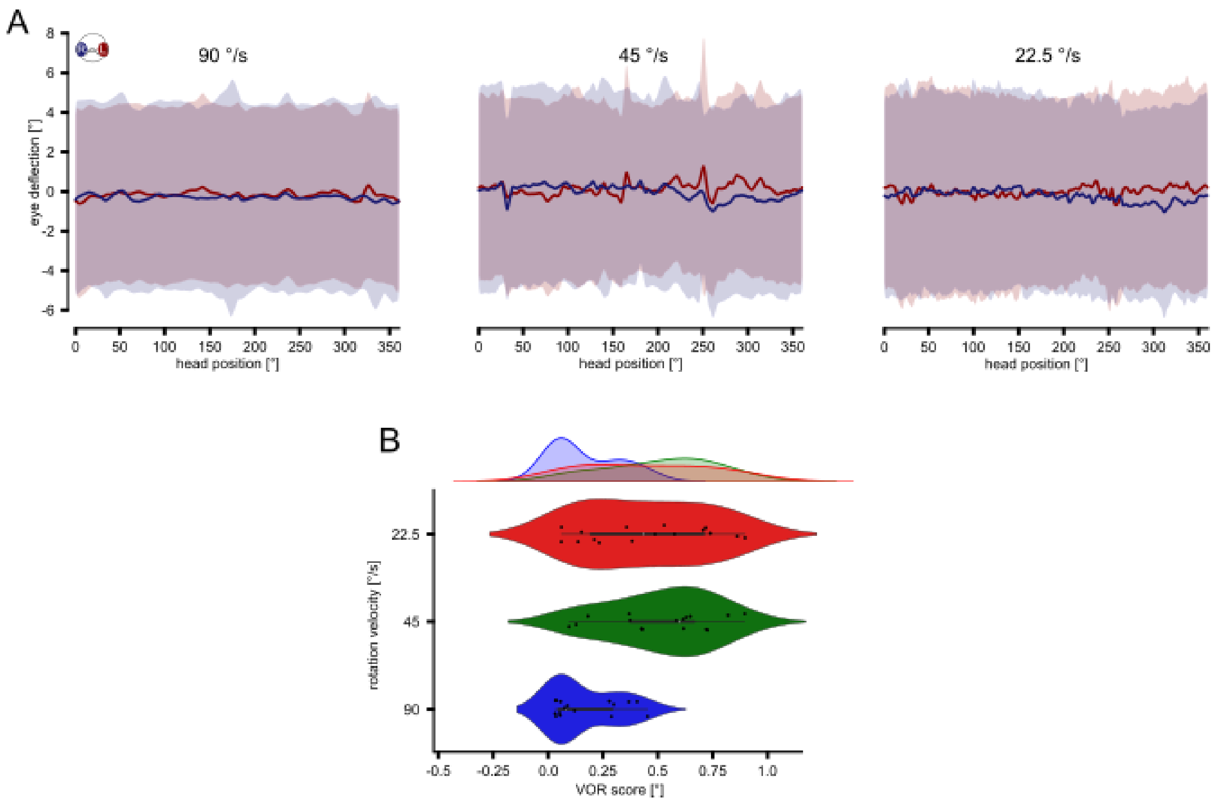


**Fig. S7.** Noise measurements. Measurements of anesthetized 4dpf larvae (168mg/l MS222) running the same VOR protocol as used in our main study. A) STA of eye movements during different rotation velocities (90 °/s, 45 °/s, 22.5 °/s). B) VOR score of anesthetized larvae for different rotation velocities (90 °/s, 45 °/s, 22.5 °/s).
